# Toll-like receptor 4 regulates intestinal fibrosis via cytokine expression and epithelial-mesenchymal transition

**DOI:** 10.1038/s41598-020-76880-y

**Published:** 2020-11-16

**Authors:** Yu Kyung Jun, So Hyun Kwon, Hee Tae Yoon, Hyunsun Park, Hosim Soh, Hyun Jung Lee, Jong Pil Im, Joo Sung Kim, Ji Won Kim, Seong-Joon Koh

**Affiliations:** 1 Department of Internal Medicine and Liver Research Institute, Seoul National University College of Medicine, 101 Daehak-ro, Jongno-gu, Seoul, 03080 Republic of Korea; 2 Division of Gastroenterology, Department of Internal Medicine, Boramae Medical Center, 20 Boramae-ro-5-gil, Dongjak-gu, Seoul, 07061 Republic of Korea; 3 Department of Dermatology, Boramae Medical Center, 20 Boramae-ro-5-gil, Dongjak-gu, Seoul, 07061 Republic of Korea; 4 Laboratory of Intestinal Mucosa and Skin Immunology, Seoul National University College of Medicine, 101 Daehak-ro, Jongno-gu, Seoul, 03080 Republic of Korea

**Keywords:** Immunology, Gastroenterology

## Abstract

Intestinal fibrosis induced by chronic and recurrent colitis, which is exacerbated by bowel stenosis, stricture, and obstruction, is challenging to treat. Toll-like receptor 4 (TLR4) stimulates innate and acquired immunity in response to specific microbial components, but the role of TLR4 in intestinal fibrosis is largely unknown. We investigated its role in intestinal fibrosis using not only a murine fibrosis model but also human myofibroblasts and intestinal epithelial cells. Colon fibrosis was induced in TLR4-deficient (TLR4^−/−^) mice and its wild-type counterparts with 3% dextran sulfate sodium. Absence of TLR4 gene attenuated chronic inflammation and colonic macrophages infiltration; intestinal fibrosis and collagen deposition were suppressed. Also, the production of tumor necrosis factor-α, interleukin-12p40, and transforming growth factor-β was reduced in TLR4-deficient peritoneal macrophages. TLR4 was silenced in CCD-18Co cells by small interfering RNA (siRNA), and matrix metalloproteinase-1, tissue inhibitor of metalloproteinase, and collagen α1 expression was evaluated. Role of TLR4 in epithelial-mesenchymal transition (EMT) was evaluated in HCT116 cells. Suppression of TLR4 transcription by siRNAs affected myofibroblasts activity, collagen synthesis, and EMT in the human cancer cell line. Thus, we suggest that TLR4 can be an essential mediator in intestinal chronic inflammation and fibrosis, indicating that TLR4 signaling is a potential therapeutic target for intestinal fibrosis.

## Introduction

Intestinal fibrosis is a chronic progressive process involving excessive deposition of extracellular matrix (ECM) components in the intestinal wall. Fibrosis develops as a result of failed wound healing in the mucosa during inflammatory responses. Multiple immune cells, including macrophages and neutrophils contribute to tissue injury by releasing oxygen radicals, cathepsin, and matrix metalloproteinase (MMP)^1^. As a counterpart, myofibroblasts, activated by inflammation, contract wound area, and produce ECM components. In chronic and recurrent inflammation, the inflammatory cascade is activated for extended time period and the negative feedback, which terminates proliferative and fibrotic response, is not enough to regulate the physiological balance between fibrosis and regeneration.

Epithelial-mesenchymal transition (EMT) is a physiological process responsible for not only intestinal fibrosis but also embryogenesis, organ development, and carcinogenesis^2,3^. During EMT, existing epithelial cells lose their polarity and become mesenchymal cells both functionally and morphologically. Interestingly, these cells can possess both epithelial markers such as E-cadherin or cytokeratin 8 and 20, and mesenchymal markers, such as α-smooth muscle actin (α-SMA) or vimentin^4^. Thus, fibrosis results in the collapse of normal tissue architecture, permanent scarring, structural change, and organ malfunction^5^.

Intestinal fibrosis can occur as a long-term complication of numerous diseases such as inflammatory bowel disease (IBD), radiation enterocolitis, chronic ischemic enterocolitis, collagenous colitis, eosinophilic enteropathy, drug-induced enteropathy, and cystic fibrosis^6^. In IBD, it is manifests as complications, including stenosis, stricture, and obstruction. Bowel stenosis and stricture provoke abnormal bowel movement and result in abdominal pain, nausea, vomiting, anorexia, and weight loss in patients with IBD. About 50% of patients with Crohn’s disease (CD) suffered from serious intestinal fibrosis that required surgery during a 10-year disease course^7^. Even after surgery, the relapse rate of intestinal fibrosis in patients with CD was too high to ignore. Patients with ulcerative colitis (UC) experience fewer intestinal fibrosis-related complications than patients with CD. The prevalence of stricture in patients with UC was reported various outcomes which were 11.2%^8^, 5%^9^, or 3.2%^10^, respectively. Therefore, the early detection and prevention of intestinal fibrosis are important to improve prognosis of patients with IBD.

Clinically-used anti-inflammatory agents to treat IBD cannot prevent the intestinal fibrosis once excessive ECM deposition process begins^11^. Although some studies reported infliximab to be a treatment for small bowel stenosis in patients with CD, the sample size was small and the treatment was far less effective than endoscopic treatment or surgery^12,13^. Moreover, patients with fibrostenotic CD, even subclinical, were more likely to be non-response to infliximab treatment, and they had to eventually undergo surgery^14^. Therefore, development of new anti-fibrotic agents for IBD is urgently required.

Damage-associated molecular patterns (DAMPs) are host-derived molecules released by damaged or dead cells^15,16^. Pathogen-associated molecular patterns (PAMPs) are microbe-derived molecular compounds, including lipopolysaccharide (LPS), flagellins, fungal glucans, bacterial DNA, and double-strand RNA^17^. DAMPs and PAMPs provoke inflammatory responses through activating pattern recognition receptors such as Toll-like receptors (TLRs). TLRs consist of TLR1 to TLR9 and induce the activation and maturation of innate and acquired immunity^18^. TLR4 is one of the TLRs and can be activated by Gram-negative-derived exogenous LPS and endogenous DAMP ligands^19^. TLR4 is expressed on intestinal epithelial cells (IECs), macrophages, and dendritic cells. The TLR4 signaling pathways mainly consist of myeloid differentiation primary response gene 88 (MyD88)-dependent pathway which stimulates nuclear factor-κB (NF-κB), members of the mitogen-activated protein kinase (MAPK) pathway, interleukin (IL)-1 pathway, and produces multiple pro-inflammatory cytokines^20^. In addition, TLR4 establishes MyD88-independent pathway responsible for late activation of NF-κB^21^. Although its contribution of intestinal inflammation is well known, the role of TLR4 in intestinal fibrosis remains unclear. Therefore, we investigated the role of TLR4 in intestinal inflammation and thereby the importance in intestinal inflammation as well as fibrosis.

## Results

### TLR4-deficiency attenuated the severity of chronic inflammation and fibrosis in murine model of colitis

**Figure 1:**
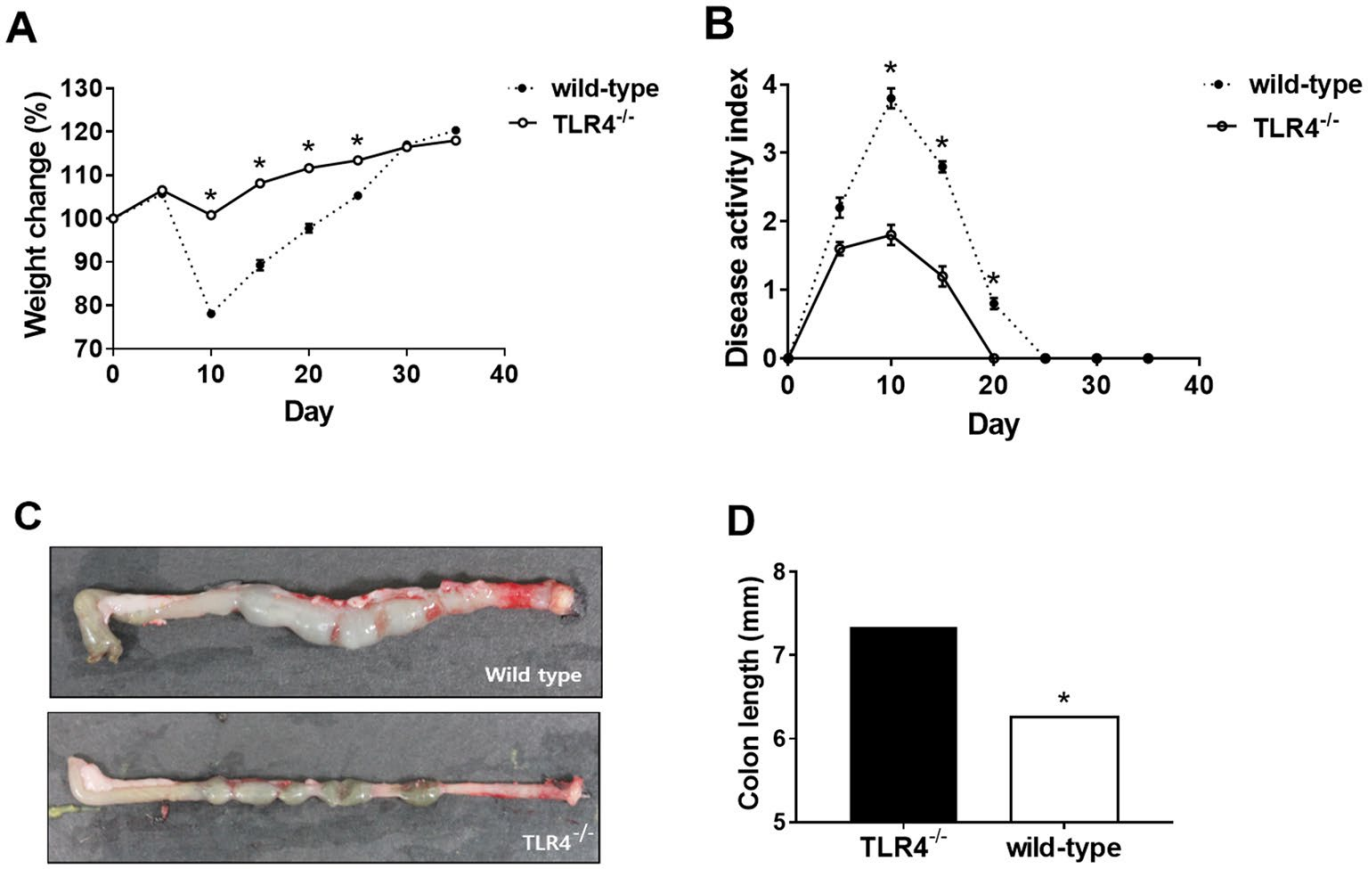
Comparison of clinical indices, gross appearance, and histologic grading in toll-like receptor 4-deficient (TLR4^−/−^) (n = 5) and wild-type mice (n = 5). (**A**) Wild-type mice experienced extensive weight loss compared to TLR4^−/−^ mice during post dextran sulfate sodium treatment. (**B**) Disease activity index from day 5 to day 20 was higher in wild-type as compared to TLR4^−/−^ mice. (**C**) Gross appearance of extracted colons showed bowel edema and fibrotic change in wild-type mice. However, in TLR4^−/−^ mice, bowel edema was not marked. (**D**) Colon from TLR4^−/−^ mice was longer than that from wild-type mice. Asterisks indicate significant differences (*P*-values < 0.05) between TLR4^−/−^ and wild-type mice.

To evaluate the role of TLR4 in chronic colon inflammation and fibrosis, we studied a dextran sulfate sodium (DSS)-induced murine chronic colitis model. We analyzed phenotypic characteristics of the DSS-administered mice, such as body weight and disease activity index (DAI) every five days. Wild-type mice showed significantly greater weight reduction and higher DAI as compared to TLR4^−/−^ mice (Fig. 1A,B); while the weight of wild-type mice rapidly decreased, TLR4^−/−^ mice did not show definite weight loss. Wild-type mice had higher DAI than TLR4^−/−^ mice from day 5 to 20, and both groups recovered to score zero after day 20. The colon samples from wild-type mice showed higher degree of bowel wall edema and fibrotic changes (Fig. 1C). Unlike wild-type mice, solid feces were detected in TLR4^−/−^ mice colon. The colon length of wild-type mice was significantly reduced as compared to that of TLR4^−/−^ mice (Fig. 1D). These results indicated that TLR4 deletion alleviated colitis and fibrotic changes, suggesting that it may aggravate chronic colon inflammation and fibrosis.

### TLR4 induced collagen deposition in colon submucosa along with colonic infiltration of macrophages and myofibroblasts

**Figure 2:**
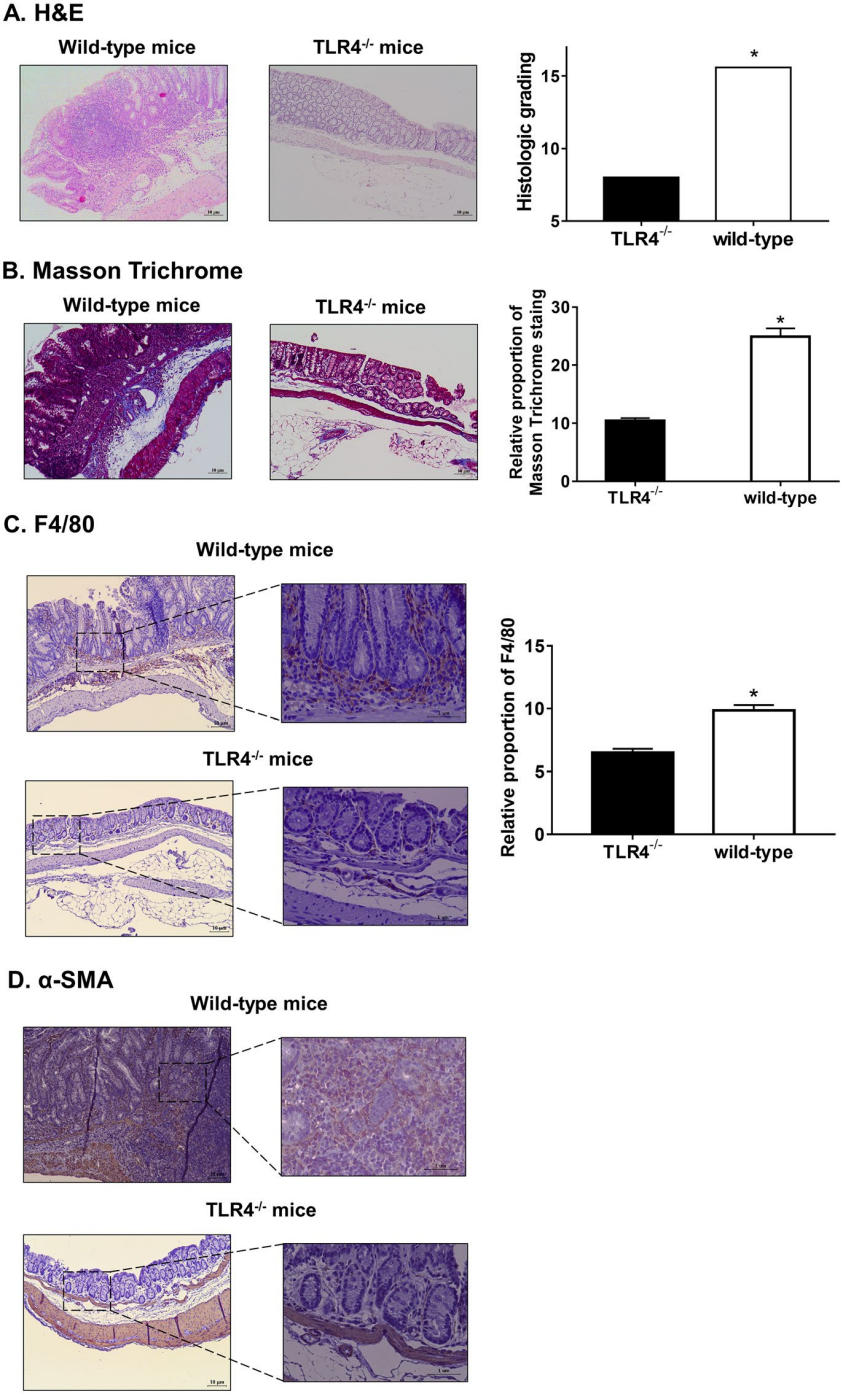
Representative hematoxylin–eosin (H&E) staining, Masson’s Trichrome (MT) staining, and immunohistochemistry. (**A**) H&E staining revealed prominent mucosal disruption and infiltration of inflammatory cells in wild-type colon. (magnification: × 100). Histological grading, determined by H&E staining, was higher in wild-type than TLR4^−/−^ mice. (**B**) MT staining of colon tissue samples revealed more severe fibrosis in wild-type than in TLR4^−/−^ mice (magnification: × 100). The proportion of MT-positive area, analyzed using ImageJ program, was significantly lower in TLR4^−/−^ than in wild-type colon tissue. (**C**) Immunohistochemistry detected increased migration of F4/80-positive cells in wild-type, as compared to TLR4^−/−^ mice (magnification: × 100 and × 400). The proportion of F4/80-positive region was markedly lower in TLR4^−/−^ as compared with wild-type mice. (**D**) The proportion of α-smooth muscle actin (α-SMA)-positive cells was higher in distorted mucosal structure of wild-type than TLR4^−/−^ mice (magnification: × 100 and × 400). Statistically significant results: *P-values < 0.05.

The severity of colitis and intestinal inflammation was assessed by histopathological analysis using hematoxylin–eosin (H&E) staining (Fig. 2A). Histopathological examination revealed severe inflammation and fibrosis in the colon of wild-type mice exposed to DSS. Distorted crypt architecture, multiple inflammatory cells, and extensive edema in the mucosa and submucosa were detected. In contrast, TLR4^−/−^ mice exposed to DSS showed well-preserved mucosal integrity. Additionally, the histological grades between the two groups were markedly different. Masson’s Trichrome (MT) staining of collagen within the colon tissue revealed suppressed collagen deposition in the submucosa of TLR4^−/−^ mice, and showed severe colon fibrosis in wild-type mice (Fig. 2B). Next, to determine macrophage and myofibroblast distribution in the colon, we performed immunohistochemical staining using respective markers, F4/80 and α-SMA (Fig. 2C,D). We noted that a higher number of F4/80- or α-SMA-positive cells infiltrated the lamina propria in wild-type mice as compared to that in TLR4^−/−^ mice.

### Intestinal fibrosis might promote the expression of tumor necrosis factor-α (TNF-α) and IL-12p40 in LPS-treated murine peritoneal macrophages

**Figure 3:**
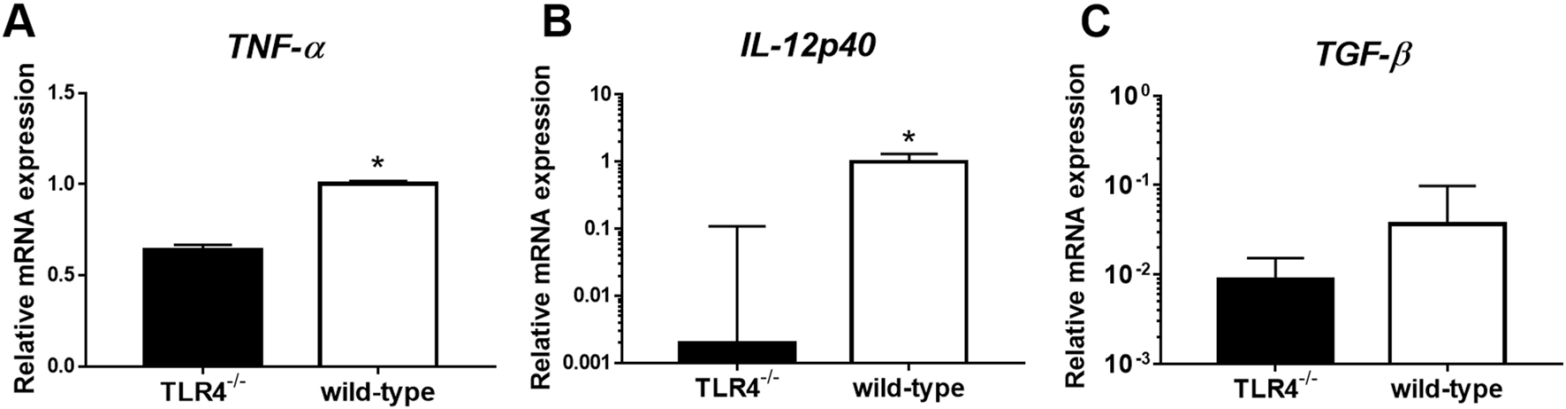
Comparison between the expression levels of inflammatory cytokines in peritoneal macrophages obtained from TLR4^−/−^ mice (n = 3) and wild-type mice (n = 3) after lipopolysaccharides (LPS) stimulation (10 ng/mL for four hours). The transcription levels of tumor necrosis factor-α (TNF-α), interleukin (IL)-12p40, and transforming growth factor-β (TGF-β) were measured by real-time reverse transcription-polymerase chain reaction (RT-PCR). The results are representative data of three separate experiments. Asterisks indicate significant differences (*P*-values < 0.05) between two groups.

To assess the role of TLR4 and elucidate the mechanisms responsible for intestinal fibrosis, the transcription levels of TNF-α, IL-12p40, and transforming growth factor-β (TGF-β) were investigated in LPS-treated peritoneal macrophages from TLR4^−/−^ and wild-type mice (Fig. 3A–C). LPS stimulation resulted in reduced transcription levels of TNF-α and IL-12p40 in peritoneal macrophages from TLR4^−/−^ mice. The transcription levels of TNF-α and IL-12p40 in macrophages from LPS-stimulated TLR4^−/−^ and wild-type mice were significantly different. Otherwise, TGF-β expression was not shown any statistical significance.

### TLR4 facilitated the expression of tissue inhibitor of metalloproteinase (TIMP) and collagen α1 in human colon myofibroblasts

**Figure 4:**
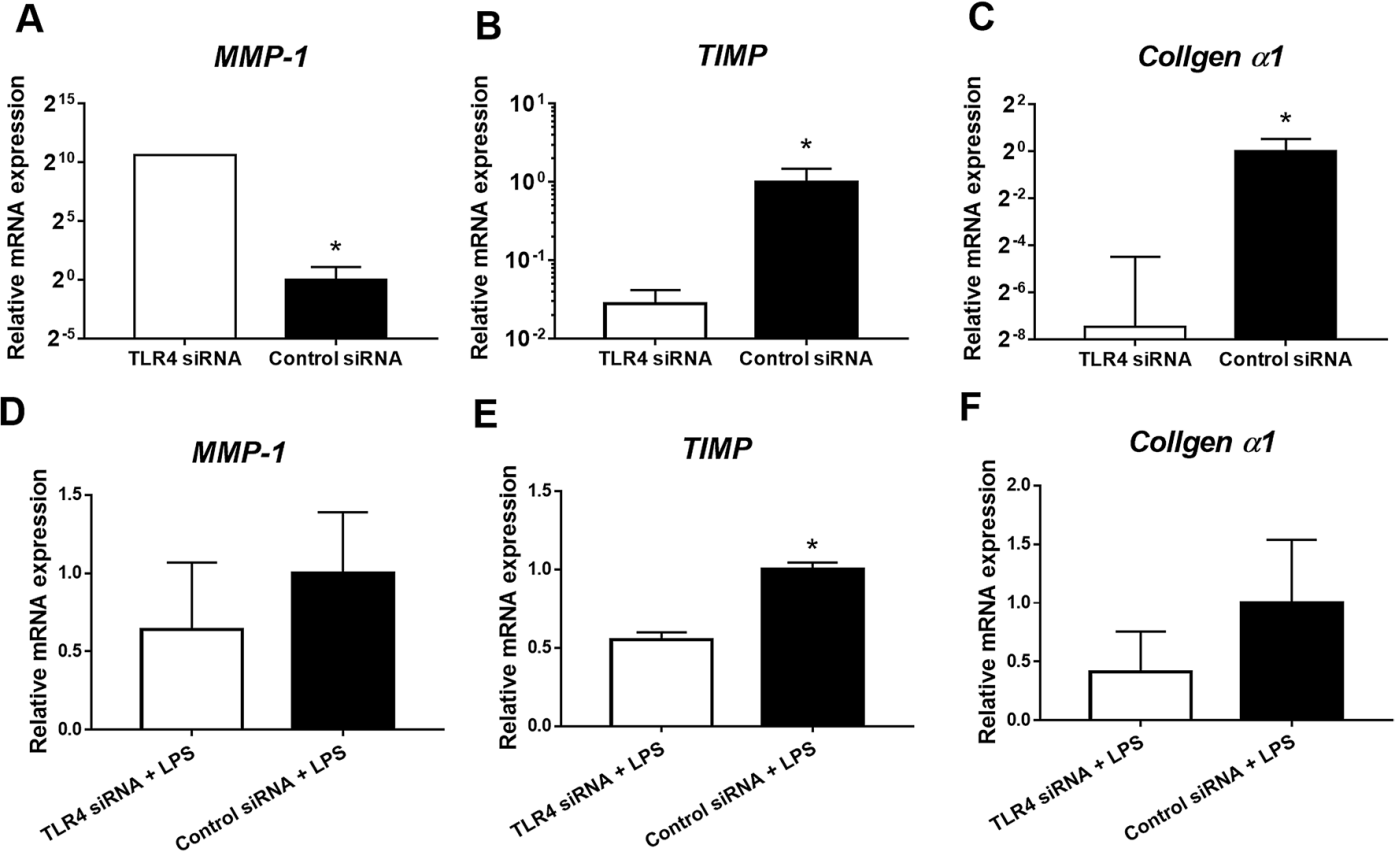
The effect of TLR4 silencing on gene expression in human colon myofibroblasts. Transfection with TLR4 small interfering RNA (siRNA) (n = 5) or control siRNA (n = 5) with or without LPS (10 ng/mL for four hours) stimulation was performed in CCD-18Co cells. The transcription levels of matrix metalloproteinase-1 (MMP-1), tissue inhibitor of metalloproteinase (TIMP), and collagen α1 were evaluated by real-time RT-PCR. The expressions of TIMP and collagen α1 were decreased when TLR4 siRNA was treated regardless LPS stimulation. The expression of MMP-1 increased when TLR4 siRNA was treated without LPS, but it decreased when LPS was administrated. The results are representative data of three separate experiments. Statistically significant results: *P-values < 0.05.

Intestinal inflammation play a key role in fibrotic process^22^. However, recent studies suggest that fibrotic mechanisms can be distinct from intestinal inflammation^23^. To confirm the role of TLR4 signaling in colon myofibroblasts, we investigated the effect of TLR4 silencing with or without LPS stimulation on the activation of myofibroblasts. TLR4 knockdown without LPS stimulation in CCD-18Co cells resulted in increased MMP-1 expression as compared to those transfected with control small interfering RNA (siRNA) (Fig. 4A). However, transfection with TLR4 siRNA significantly suppressed the expression of TIMP (Fig. 4B), and collagen α1 (Fig. 4C). When stimulating with LPS in CCD-18Co cells, there was no significant difference in the expression of MMP-1 (Fig. 4D). However, similar results were shown in TIMP and collagen α1 expression. (Fig. 4E, and 4F). This indicated that TLR4 is an essential for TIMP expression and collagen production in myofibroblasts.

### TLR4 modulated EMT in human IECs

**Figure 5:**
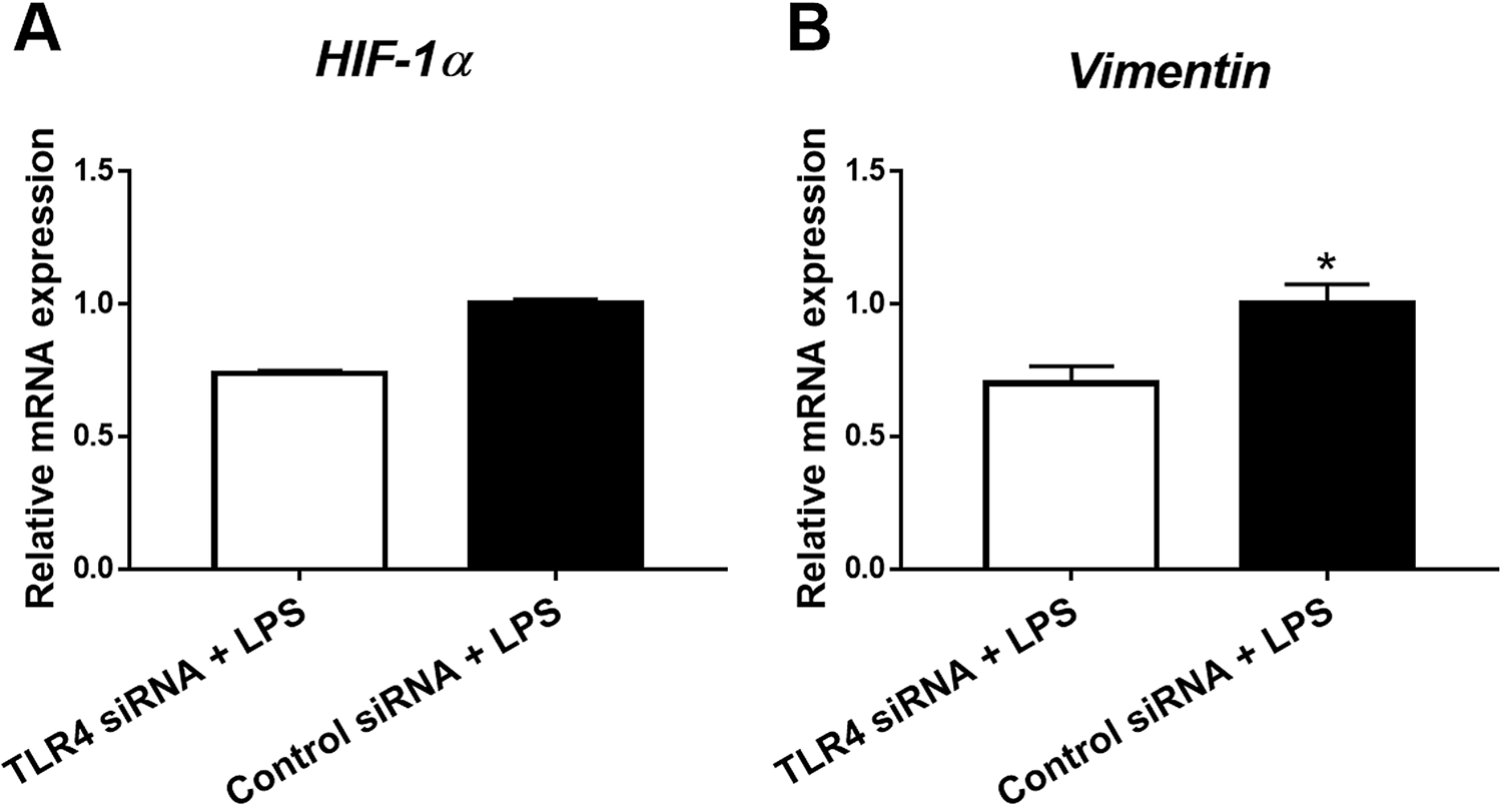
The effect of TLR4 silencing on epithelial-mesenchymal transition in HCT116 cells. HCT116 cells were transfected with TLR4 siRNA (n = 5) or control siRNA (n = 5). After TLR4 silencing, LPS (10 ng/mL for four hours) was treated. The gene expression levels of vimentin were suppressed. The results are representative data of three separate experiments. Statistically significant results: *P-values < 0.05.

Previously, hypoxia-inducible factor-1α (HIF-1α) and vimentin have been known as mesenchymal markers^24,25^. Therefore, we investigated whether TLR4 signaling facilitated EMT in IECs (Fig. 5A,B). TLR4 silencing with LPS stimulation in CCD-18Co cells resulted in significant suppression of vimentin expression.

## Discussion

Our current study is the first to demonstrate that TLR4 may have an essential function in not only intestinal inflammation but also intestinal fibrosis. In a DSS-induced murine chronic colitis model, the genetic deletion of TLR4 strongly suppressed colon inflammation and fibrosis. In LPS-stimulated peritoneal macrophages, the expression of TNF-α and IL-12p40 was decreased in TLR4^−/−^ mice as compared to that in wild-type mice. The silencing of TLR4 through siRNA transfection weakened the activation of human myofibroblasts and collagen synthesis. In addition, TLR4 might regulate EMT in human IECs. To our knowledge, this is the first study to demonstrate the role of TLR4 in colon fibrosis.

TLR4, stimulated by Gram-negative bacteria-produced LPS or endogenous ligand-produced by damaged host cells, can facilitate inflammatory responses through the maturation of innate immunity^26,27^. Further, TLR4 has a potential function in IBD development; the frequency of TLR4 Asp299Gly polymorphism was higher in patients with UC, and TLR4 expression was increased in the intestine of patients with active UC^28^. In the present study, TLR4 regulated not only chronic colitis but also fibrosis. TLR4 exacerbated collagen deposition in colon wall along with macrophages and myofibroblasts infiltration into colon. Taken together, we believe that TLR4 signaling contributes colon fibrosis as well as chronic inflammation, TLR4 signaling pathway is a potential target for the prevention of colon fibrosis in IBD.

During chronic colitis and tissue injury, macrophages contribute to the development of intestinal fibrosis^29,30^. M1 macrophages produce pro-inflammatory cytokines such as IL-1β and TNF-α, which initiate fibrotic processes. M2 macrophages produce pro-fibrotic cytokines such as TGF-β, connective tissue growth factor, platelet-derived growth factor, fibroblast growth factor, and insulin-like growth factor^31^. TNF-α produced by macrophages can facilitate mucosal remodeling and fibrosis^32^. TNF-α-stimulated macrophages promote production of TGF-β1 and MMP in myofibroblasts^33^. Synergic effect between TNF-α and TGF-β also promote EMT^34^. IL-12p40 favors M1 polarization and can induce inflammation and fibrosis^35^. In the present study, TLR4 was critical for peritoneal macrophages to activate signaling cascade through TNF-α and IL-12p40 expression. However, there was no significant difference on the expression of TGF-β between peritoneal macrophages from wild-type and TLR4^−/−^ mice. Based on these, we suggest that TLR4 signaling may play a role in colon fibrosis by M1 macrophages rather than M2 macrophages.

The role of TLR4 signaling on the expression of MMPs remains obscure. Previously, TLR4 activation by LPS leads to increased MMP-1 expression in small airway cells and nasal polyp-derived fibroblasts^4,5^. However, a previous study has shown that MMP-2 expression was not affected by LPS-induced TLR4 activation in corneal fibroblasts^36^. In our study, knockdown of TLR4 without LPS stimulation resulted in significant suppression of MMP-1 expression in CCD-18Co cells. However, silencing of TLR4 was not associated with the increased expression of MMP-1, suggesting the effect of TLR4 signaling on the expression of MMPs might depend on the cell types or culture environment. Under homeostatic conditions, MMPs are constitutively expressed at a low level and are closely regulated by TIMPs. In damaged tissues, myofibroblasts facilitate TIMP-1 production that inhibits MMPs and may thus cause enhanced deposition of ECM proteins, contributing to intestinal fibrosis^37,38^. Our results demonstrated that silencing of TLR4 was associated with the increased expression of TIMP regardless of LPS stimulation. Based on these results, TLR4 signaling-induced TIMP-1 expression in colon myofibroblasts may play an important role in the process of intestinal fibrosis. More studies are required for demonstrating the role of MMP-1 in intestinal fibrosis.

Studies have reported that mesenchymal stem cells migrate to the damaged intestine in response to the homing signal generated by chemotactic factors such as TGF-β1^39,40^. However, myofibroblasts may be derived from non-mesenchymal origins, including epithelial cells via EMT. We found reduced number of myofibroblasts in the colon tissues from TLR4^−/−^ mice. Therefore, we further evaluated the effect of TLR4 silencing on the expression of EMT-related genes in HCT116 cells. Our study revealed that TLR4 might promote EMT pathway in HCT116 cells via activation of vimentin. Therefore, we suggest that TLR4 signaling pathway may contribute to EMT, thereby regulating intestinal fibrosis. To our knowledge, this is the first study to demonstrate the role of TLR4 in cell migration associated with intestinal fibrosis.

In conclusion, findings of the present study indicated that TLR4 might act as a key mediator in intestinal inflammation and fibrosis by regulating cytokines expression on intestinal macrophages and myofibroblasts. Therefore, TLR4 signaling pathway may be a potential treatment target for intestinal fibrosis and help patients with chronic colitis prevent or overcome fibrostenosis.

## Methods

### Mice

TLR4^−/−^ mice with C57BL/6 background aged 7 to 8 weeks were used. Age- and gender-matched wild-type mice (C57/6NCrljBgi), purchased from Orient (Seongnam, Korea), were used as control. The mice were maintained under specific pathogen-free housing with standard conditions of humidity, temperature, and a light/dark cycle in the Laboratory of Experimental Animal Research of Boramae Medical Center. Mice were euthanized with isoflurane inhalation either on day or early, when mice exhibited severe body weight loss (25% of their pre-experimental body weight), according to the protocol.

### Induction and evaluation of DSS-induced colon fibrosis

DSS (MP Biochemical, Irvine, CA, USA), a water-soluble, negatively charged sulfated polysaccharide, can cause intestinal inflammation. To induce colon fibrosis, 3% DSS dissolved in drinking water was administrated for five days^41^. Five mice were randomly assigned to each group. It has already been proven that control group treated with normal water without DSS did not develop chronic fibrotic colitis^42,43^. Therefore, negative control groups with normal water were not assigned in our experiments due to ethical concerns. Mice were assessed every five days for behavior, water/chow consumption, body weight, stool consistency, and evidence of gross hematochezia. DAI for assessment of colitis severity was determined as the sum of parameters, consisting of the changes in body weight loss, stool consistency, and presence of rectal bleeding^44^. Weight loss was calculated as the percent difference between the original weight (day 0) and the weight on any particular day.

On day 33, colon tissue samples were obtained under anesthesia, fixed in 10% buffered formalin, and embedded in paraffin. Sections were stained with H&E. The severity of colitis was scored by two examiners not involving experimental procedures as described previously^45^. In brief, three independent parameters were measure: inflammation severity (0, none; 1, mild; 2, moderate; 3, severe), inflammation extent (0, none; 1, mucosa; 2, mucosa and submucosa; 3, transmural), the extent of crypt damage (0, none; 1, damage to the basal one-third portion; 2, damage to the basal two-thirds portion; 3, damage to the entire crypt with surface epithelium intact; 4, erosion). Sum of these scores were quantified as to the percentage of tissue involvement (0, 0%; 1, 1–25%; 2, 26–50%; 3, 51–75%; 4, 76–100%). To identify intestinal fibrosis, colon tissue samples were stained with MT. The difference in location and extent of positive area in MT-stained tissues were analyzed under an optical microscope and measured as the percent positive area using ImageJ software (available at https://rsbweb.nih.gov/ij/). The average of three regions from each slide was considered.

### Immunohistochemical staining of mouse colon tissues

Immunohistochemistry for F4/80 and α-SMA, markers for macrophages and myofibroblasts, respectively, was performed on colon tissue samples as previously described^46^. Thin slices obtained from the formalin-fixed paraffin-embedded colon tissue blocks were stained. Paraffin wax was removed from the samples and the samples were rehydrated. The slides were then immersed three times in a citrate buffer (pH 6.0), maintained just below the boiling temperature for five minutes each, and then cooled for 20 minutes. After washing with Tris-buffered saline (TBS) containing 0.025% Triton X-100, the slides were stained with primary F4/80 antibody (Abcam, Cambridge, MA, USA) or α-SMA antibody (Abcam). The slides were washed with TBS containing 0.025% Triton X-100 and incubated with a secondary antibody. After developing with a chromogen, the slides were counterstained using hematoxylin. Immunoreactivity of F4/80 was measured as the percent positive area using ImageJ software program. More than five areas encompassing both mucosa and submucosa were evaluated for each slide.

### Isolation of murine peritoneal macrophages

We extracted murine peritoneal macrophages as described previously^47–49^. Briefly, mice were intraperitoneally injected with 2 mL of 4% thioglycolate (Sigma-Aldrich, St. Louis, MO, USA). After four days from the injection, 10 ml of Hank’s balanced salt solution (Corning Cellgro, Manassas, VA, USA) was intraperitoneally injected at the site and extracted with a syringe. The collected peritoneal fluid was centrifuged, and cells were resuspended in Roswell Park Memorial Institute (RPMI)-1640 medium (Invitrogen, Carlsbad, CA, USA). After culturing for two hours, cells were washed, and the remaining cells were collected as peritoneal macrophages.

To elucidate mechanisms responsible for intestinal fibrosis, isolated peritoneal macrophages were exposed to bacterial endotoxin, LPS. TLR4^−/−^ peritoneal macrophages and their wild-type counterparts were pretreated with sulforaphane and challenged with 10 ng/mL LPS (Sigma-Aldrich). After four hours of LPS stimulation, the transcription levels of inflammatory cytokines were measured through real-time reverse transcription-polymerase chain reaction (RT-PCR).

### Culture and preparation of human cell lines

The human colon myofibroblasts, CCD-18Co cells (Korean Cell Line Bank 21,459, Seoul, Korea) were cultured in a Dulbecco's modified Eagle medium (DMEM)/Earle's balanced salt solution (EBSS) medium supplied with 10% fetal bovine serum (FBS), 1 mM non-essential amino acids, 1 mM sodium pyruvate, and 2 mM sodium bicarbonate without antibiotics. HCT116 human IECs (Korean Cell Line Bank 10,247, Seoul, Korea) were cultured in the RPMI-1640 medium containing 5% FBS. All cells were incubated at 37 °C in a humidified 5% CO_2_ incubator.

TLR4 was silenced by transfecting CCD-18Co cells or HCT116 cells with siRNAs specific for human TLR4 (Santa Cruz Biotechnology, Santa Cruz, CA, USA). TLR4 siRNAs were transfected using WelFect-EX reagents (Welgene, Daegu, Korea) as per the manufacturer’s instructions. Scrambled siRNAs (Santa Cruz Biotechnology) were used as controls. After transfections, CCD-18Co cells were treated with or without 10 ng/mL LPS for four hours. HCT116 cells were treated with 10 ng/mL LPS after transfections.

### RT-PCR

The role of TLR4 in LPS-treated murine peritoneal macrophages was assessed by evaluating the expression of TNF-α, IL-12p40, and TGF-β by real-time RT-PCR. Total RNA was isolated from murine peritoneal macrophages, and complementary DNA (cDNA) was synthesized using amfiRivert cDNA Synthesis Platinum Master Mix (GenDEPOT, Texas, USA) from total RNA isolated from peritoneal macrophages. The primers used were as follows: TNF-α, (5′-CAT CTT CTA AAA ATC GAG TGA CAA-3′ and 5′-TGG GAG TAG ACA AGG TAC AAC CC-3′); IL-12p40, (5′-GGA AGC ACG GCA GCA GAA TA-3′ and 5′-AAC TTG AGG GAG AAG TAG GAA TGG-3′); TGF-β, (5′-TGG AAA TCA ACG GGA TCA G-3′ and 5′-GTC CAG GCT CCA AAT ATA GG-3′). The relative changes in gene expression were calculated by normalizing the expression level of the target gene to the level of Glyceraldehyde 3-phosphate dehydrogenase (GAPDH).

Similarly, the expressions of MMP-1, TIMP, and collagen α1 in CCD-18Co cells treated with TLR4 siRNA, and those of HIF-1α and vimentin in HCT116 cells were also evaluated by real-time RT-PCR. The primers were as follows: MMP-1, (5′- GGT GAT GAA GCA GCC CAG-3′ and 3′- CAG TAG AAT GGG AGA GTC-5′); TIMP, (5′- ART CAA CSA GAC CAC CTT ATA CCA-3′ and 3′- ASC TGR TCC GTC CAC AAR CA-5′); collagen α1, (5′-GAA CGC GTG TCA TCC CTT GT-3′ and 3′-GAA CGA GGT AGT CTT TCA GCA ACA-5′); HIF-1α, (5′- CAT CTC CAT CTC CTA CCC ACA T-3′ and 3′- ACT CCT TTT CCT GCT CTG TTT G-5′); vimentin, (5′- GAA GAG AAC TTT GCC GTT GAA G-3′ and 3′- ACG AAG GTG ACG AGC CAT T-5′).

### Statistical analysis

Data are expressed as mean and standard error of the mean (SEM). Non-parametric Mann–Whitney test was performed to compare values between the groups using SPSS 25 statistical software (SPSS, Chicago, IL, USA). The *P*-values less than 0.05 were considered statistically significant.

### Ethics approval

All procedures were approved by the Animal Care Committee at SMG-SNU Boramae Medical Center (IACUC No.2016–0017). All animal experiments were conducted in accordance with the Guide for the Care and Use of Laboratory Animals published by the National Institute of Health.
